# PSA-density, DRE, and PI-RADS 5: potential surrogates for omitting biopsy?

**DOI:** 10.1007/s00345-024-04894-6

**Published:** 2024-03-20

**Authors:** Fabian Falkenbach, Francesca Ambrosini, Mykyta Kachanov, Gernot Ortner, Tobias Maurer, Daniel Köhler, Dirk Beyersdorff, Markus Graefen, Lars Budäus

**Affiliations:** 1 Martini-Klinik Prostate Cancer Center, University Hospital Hamburg-Eppendorf, Martinistrasse 52, 20246 Hamburg, Germany; 2 Department of Urology, IRCCS Ospedale Policlinico San Martino, Genoa, Italy; 3 Institute of Human Genetics, University Medical Center Hamburg-Eppendorf, Hamburg, Germany; 4 Department of Urology, University Medical Center Hamburg-Eppendorf, Hamburg, Germany; 5 Department for Radiology and Nuclear Medicine, University Medical Center Hamburg-Eppendorf, Hamburg, Germany

**Keywords:** Prostate cancer, mpMRI, Image-guided biopsy, PI-RADS, DRE, PSA-density

## Abstract

**Objective:**

In contrast to other malignancies, histologic confirmation prior treatment in patients with a high suspicion of clinically significant prostate cancer (csPCA) is common. To analyze the impact of extracapsular extension (ECE), cT-stage defined by digital rectal examination (DRE), and PSA-density (PSA-D) on detection of csPCA in patients with at least one PI-RADS 5 lesion (hereinafter, “PI-RADS 5 patients”).

**Materials and methods:**

PI-RADS 5 patients who underwent MRI/Ultrasound fusion biopsy (Bx) between 2016 and 2020 were identified in our institutional database. Uni- and multivariable logistic-regression models were used to identify predictors of csPCA-detection (GGG ≥ 2). Risk models were adjusted for ECE, PSA-D, and cT-stage. Corresponding Receiver Operating Characteristic (ROC) curves and areas under the curve (AUC) were calculated.

**Results:**

Among 493 consecutive PI-RADS 5 patients, the median age and PSA was 69 years (IQR 63–74) and 8.9 ng/ml (IQR 6.0–13.7), respectively. CsPCA (GGG ≥ 2) was detected in 405/493 (82%); 36/493 patients (7%) had no cancer. When tabulating for PSA-D of > 0.2 ng/ml/cc and > 0.5 ng/ml/cc, csPCA was found in 228/253 (90%, PI-RADS5 + PSA-D > 0.2 ng/ml/cc) and 54/54 (100%, PI-RADS5 + PSA-D > 0.5 ng/ml/cc). Finally, a model incorporating PSA-D and cT-stage achieved an AUC of 0.79 (CI 0.74–0.83).

**Conclusion:**

In PI-RADS 5 patients, PSA-D and cT-stage emerged as strong predictors of csPCA at biopsy. Moreover, when adding the threshold of PSA-D > 0,5 ng/ml/cc, all PI-RADS 5 patients were diagnosed with csPCA. Therefore, straight treatment for PCA can be considered, especially if risk-factors for biopsy-related complications such as obligatory dual platelet inhibition are present.

**Supplementary Information:**

The online version contains supplementary material available at 10.1007/s00345-024-04894-6.

## Introduction

Some patients with a high suspicion of prostate cancer (PCA) wish to skip prostate biopsy and directly initiate PCA-treatment such as androgen deprivation therapy (ADT) or radical prostatectomy (RP). This strategy of imaging-based treatment is a common practice in other malignancies such as renal cell carcinoma [1]. Besides patients´ preferences, a subset of patients with a high suspicion of PCA is also at high-risk for biopsy-related complications such as patients with obligatory dual platelet inhibition or hematological conditions. In these cases, the added value of biopsy prior to treatment compared with biopsy-risks appear marginal.

Interestingly, Meissner et al. recently reported a small series of patients without prostate biopsy before undergoing RP. In this study, all patients harbored a PI-RADS score ≥ 4 and a PSMA-PET score ≥ 4 (on a five-point Likert scale) [2]. In accordance with the PRIMARY-trial [3], the authors concluded, that highly selected men with positive MRI and intense PSMA-uptake may avoid confirmatory biopsy, but prospective research is warranted. However, PSMA-PET/CT is not recommended for the primary diagnosis of PCA in current guidelines [4–6], related to high costs and is not widely available.

In contrast to PSMA-PET/CT, digital rectal examination (DRE), prostate-specific antigen (PSA), and multiparametric magnetic resonance imaging (mpMRI) are available before MRI/Ultrasound fusion prostate biopsy (Bx). While MRI has a high negative predictive value for clinically significant PCA (csPCA, GGG ≥ 2) [4], within the PRECISION trial [7], 6% of patients with PI-RADS 5 lesions harbored no PCA at biopsy. These findings are consistent with those of other trials [8]. Therefore, mpMRI alone appears to be insufficient for omitting biopsies.

PSA-density (PSA-D) is associated with high-risk of csPCA [4, 9, 10]. For instance, Washino et al. reported a 97% detection rate of csPCA in patients with PSA-D ≥ 0.3 and PI-RADS 4–5 findings [9]. In addition, positive digital rectal examination (DRE) is an independent predictor of csPCA, as clearly demonstrated in the ERSPC-trial [11].

Therefore, we analyzed in a subgroup of patients with at least one PI-RADS 5 lesion (hereinafter, named “PI-RADS 5 patients”), whether the prediction of csPCA may be easily enhanced adding base clinical parameters, such as DRE and PSA-D. Finally, we tested if biopsies could be omitted in selected cases based on these findings.

## Materials and methods

### Patients

Within our institutional database, we identified patients who had undergone MRI/Ultrasound fusion prostate biopsy (Bx) for suspected PCA between 2016 and 2020. In general, prostate biopsy was recommended according to national and international guidelines [4, 6]. The indication for mpMRI was based on clinical suspicion of PCA and was initiated by the referring physician. Targeted imaging-guided biopsy was performed in patients with a PI-RADS score ≥ 3 [12]. Based on high clinical suspicion of PCA, also PI-RADS 2 lesions were targeted in selected patients. Patients with a previous positive biopsy, prior prostate surgery or pelvic radiation therapy, and those receiving hormone therapy or 5-alpha-reductase inhibitors were excluded from the analysis (suppl. Figure 2: Consort flow diagram). The clinical T-stage, defined by the findings at DRE, was prospectively assigned by the attending urologist according to the TNM-system [4]. All patients provided informed consent for the procedure and retrospective data analysis.

### mpMRI protocol

If performed in-house, 3.0 Tesla MRI was conducted using an Ingenia system (Philips Medical System, Best, The Netherlands) with a phased-array coil, according to the common European Society of Urogenital Radiology [13] and PI-RADS v2 guideline recommendations [12, 14]. Imaging was analyzed by a dedicated uro-radiologist with > 10 years of experience in prostate-MRI [12]. Patients were assigned the highest PI-RADS scores. I.e. patients with at least one PI-RADS 5 lesion were classified as "PI-RADS 5 patients”. Extracapsular extension (ECE) was defined as overt ECE, capsular bulging or irregularity, broad capsular contact (> 1 cm), filling of the retro-prostatic angle, and asymmetry or invasion of the neurovascular bundles. If external imaging was available, second-reading by the aforementioned uro-radiologist was performed prior to biopsy.

### MRI/ultrasound fusion biopsy

Prostate biopsies were performed transrectally under oral antibiotic prophylaxis [4] and local anesthesia (peri-prostatic) by high-volume biopsy-operators (each operator performed > 300 biopsies per year). Software-assisted MRI/ultrasound fusion for targeted biopsy (TBx) was achieved using the Urostation system (Koelis, La Tronche, France). A minimum of two TBx cores were obtained from each lesion, depending on its location and size. After TBx was completed, a systematic biopsy (SBx) of the residual prostate was performed to achieve whole gland coverage including the complete apex [15]. All cores were individually sampled, documented, and analyzed.

### Outcomes of interest

The primary outcome of interest was the detection of csPCA in PI-RADS 5 patients. csPCA was defined as any prostate cancer with a Gleason Grade Group  (GGG) ≥ 2. Patients’ baseline characteristics (age, number of previous prostate biopsies, PSA level, DRE, prostate volume), biopsy parameters (number of biopsy cores taken, number of positive cores according to histopathology, detection of csPCA), and mpMRI results (maximum PI-RADS score, suspicion of ECE) were analyzed. To calculate the PSA-density (PSA-D), the serum PSA level was divided by the prostate volume measured by mpMRI, using the ellipsoid formula. All data were prospectively stored in an institutional database (FileMaker Pro 10; FileMaker Inc., Santa Clara, USA).

### Statistical analysis

Descriptive statistics included frequencies and proportions for categorical variables. Medians and interquartile ranges (IQR) were reported for continuously-coded variables. We relied on univariable and multivariable logistic-regression model analyses to test significant predictors for csPCA at biopsy and to build csPCA-predictive MRI-based risk-models in the subgroup of PI-RADS 5 patients. The covariates for adjustment were predefined and consisted of ECE (yes vs. no), PSA-D (continuously coded), and cT-stage (cT1 vs. cT2). Receiver Operating Characteristic (ROC) curves were drawn for the models, and the corresponding areas under the curve (AUC) were compared using the DeLong-test. All tests were two-sided, and the significance-level was set at p < 0.05. The R software environment for statistical computing and graphics (version 3.4.3, R Foundation for Statistical Computing) was used for all statistical analyses.

## Results

Among 3,314 consecutive patients the median age and PSA was 66 years (IQR: 60–72) and 7.3 ng/ml (IQR: 5.2–10.6; Table 1), respectively. The maximum PI-RADS scores of 3, 4, and 5 were found in 716/3,314 (22%), 2,072/3,314 (63%), and 493/3,314 (15%) patients, respectively. Median prostate volume and PSA-density was 50 cc (IQR: 35–69) and 0.15 ng/ml/cc (IQR: 0.10–0.22). Among PI-RADS 5 patients, PSA and PSA-D was 8.9 ng/ml (IQR: 6.0–13.7) and 0.2 ng/ml/cc (IQR: 0.13–0.32), respectively. Of 260/493 patients (53%) with documented measurements, the median diameter of PI-RADS 5 lesions was 19 mm (IQR: 17–23).

**Table 1:** Descriptive characteristics of 3,314 consecutive patients at prostate biopsy between 2016 and 2020, depicting for the subgroup PI-RADS 5 patients

	Overall cohort (n = 3314)	PI-RADS 5 patients (n = 493)
Age, years (median, IQR)	66 (60, 72)	69 (63, 74)
PSA, ng/mL (median, IQR)	7.3 (5.2, 10.6)	8.9 (6.0, 13.7)
Prostate volume, cc (median, IQR)	50 (35, 69)	45 (35, 60)
PSA-Density, ng/ml/cc (median, IQR)	0.15 (0.10, 0.22)	0.2 (0.13–0.32)
< 0.1	878 (26)	67 (14)
< 0.2	1427 (43)	172 (35)
< 0.5	891 (27)	200 (41)
≥ 0.5	118 (4)	54 (11)
cT-stage (n, %)		
cT1	2865 (86)	350 (71)
cT2	449 (14)	143 (29)
PI-RADS (n, %)		
2	33 (1)	
3	716 (22)	
4	2072 (63)	
5	493 (15)	493 (100)
ECE	31 (1)	31 (6)
All PCA (n, %)	2322 (70)	457 (93)
cisPCA (GGG = 1) (n, %)	644 (19)	52 (11)
csPCA (GGG > 1) (n, %)	1678 (51)	405 (82)
Biopsy-naive (n, %)	1502 (45)	314 (64)

*ECE*  extracapsular extension, *GGG*  Gleason Grade Group, *PCA*  Prostate Cancer, *cisPCA*  clinically insignificant PCA (GGG = 1), *csPCA*  clinically significant PCA (GGG ≥ 2), *PSA*  prostate-specific antigen, *mpMRI*  multiparametric prostatic MRI

Clinically significant prostate cancer (csPCA, GGG ≥ 2) was detected in 1,678/3,314 (51%) patients and in 405/493 (82%) PI-RADS 5 patients. Interestingly, no PCA was detected in 36/493 (7%) of PI-RADS 5 patients. Tabulating for different PSA-D subgroups of > 0.2 and > 0.5 ng/ml/cc within PI-RADS 5 patients, csPCA was found in 228/253 (90%) and 54/54 (100%) patients. In PI-RADS 5 patients with PSA-D between 0.2 and 0.5 ng/ml/cc, csPCA was found in 57/59 (97%) patients if DRE was positive (Table 2).

**Table 2:** Detection rates of prostate cancer within different subgroups and stratified according to Gleason Grade Groups (GGG)

	No Ca	csPCA	GGG 1	GGG 2	GGG 3	GGG 4	GGG 5	Total
PI-RADS 5	36 (7%)	405 (82%)	52 (11%)	228 (46%)	104 (21%)	11 (2%)	62 (13%)	493
PI-RADS 5 + PSA-D < 0.2	28 (12%)	176 (74%)	34 (14%)	111 (47%)	41 (17%)	3 (1%)	21 (9%)	238
PI-RADS 5 + 0.2 ≤ PSA-D < 0.5	7 (4%)	175 (88%)	18 (9%)	93 (47%)	46 (23%)	5 (3%)	31 (16%)	200
PI-RADS 5 + PSA-D ≥ 0.5	0	54 (100%)	0	24 (44%)	17 (31%)	3 (6%)	10 (19%)	54
PI-RADS 5 + PSA-D < 0.2 + pos. DRE	0	61 (92%)	5 (8%)	28 (42%)	21 (32%)	1 (2%)	11 (17%)	66
PI-RADS 5 + 0.2 ≤ PSA-D < 0.5 + pos. DRE	1 (2%)	57 (97%)	1 (2%)	21 (36%)	19 (32%)	3 (5%)	14 (24%)	59
PI-RADS 5 + PSA-D ≥ 0.5 + pos. DRE	0	18 (100%)	0	6 (33%)	7 (39%)	1 (6%)	4 (22%)	18

*No Ca*  No cancer, *csPCA*  clinically significant PCA (GGG ≥ 2), *PSA-D*  prostate-specific antigen density, *DRE*  Digital Rectal Examination

When analyzing PI-RADS 5 patients without cancer detection (36/493), the pathology revealed different non-malignant findings: Focal areas of chronic prostatitis in 26/36 (72%), chronic granulomatous prostatitis in 2/36 (6%), focal subacute prostatitis in 7/36 (19%), atypical small acinar proliferation (ASAP) in 1/36 (3%), high-grade prostatic intraepithelial neoplasia (HGPIN) in 3/36 (8%), and normal prostate tissue in 7/36 (19%) were found. Based on the detection of more than one category of non-malignant findings (e.g., combination of prostatitis and HGPIN), numbers do not add up to 100%.

**Fig. 1:**
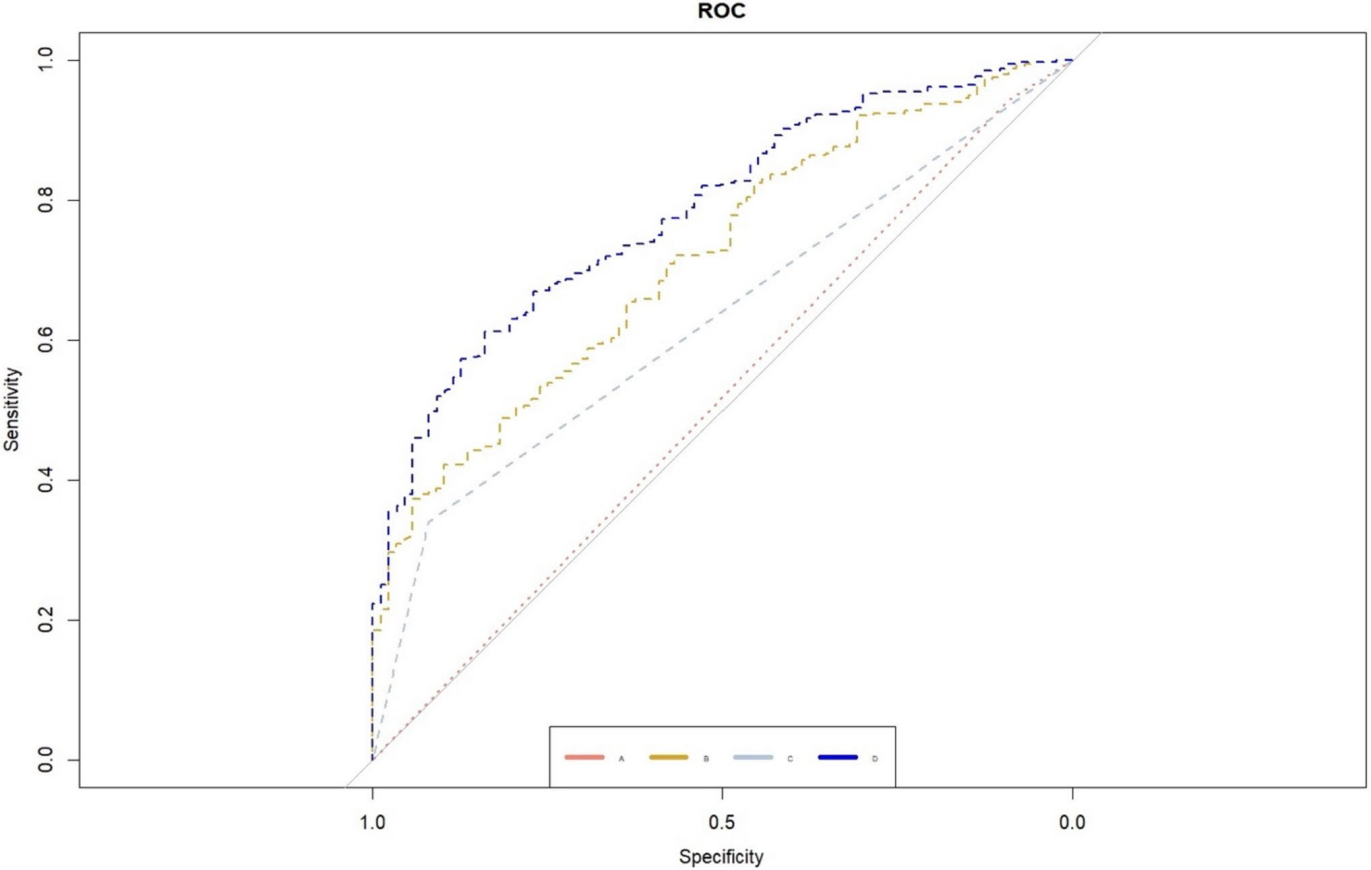
ROC curve analysis: Model A (red): ECE; Model B (yellow): PSA-density; Model C (gray): cT-stage; Model D (blue): cT-stage + PSA-density

Logistic-regression models were calculated to predict csPCA in PI-RADS 5 patients for predefined, univariable models A-C (A: ECE; B: PSA-density; C: cT-stage) (supplementary Table 3). ECE in model A did not achieve an independent predictor status (odds ratio [OR]: 0.60; 95% confidence interval [CI]: 0.27–1.48, p = 0.2). Both, PSA-density (Model B, OR: 2.12, 95% CI = 1.65–2.82, p < 0.001) and cT-stage (Model C, OR: 5.89, CI: 2.83–14.3, p < 0.001), as individual predictors, significantly increased the risk of csPCA (supplementary Table 3). Therefore, Model D, which incorporated Models B and C, was calculated. ROC curves for models A–D were generated to detect csPCA (Fig. 1). The highest AUC of 0.79 (CI: 0.74–0.83) was seen in Model D (supplementary Table 3).

## Discussion

The added value of MRI and consecutive fusion biopsies for the diagnosis of csPCA has been demonstrated in large trials [7, 8, 16]. Therefore, our study on the diagnostic influence of easy and inexpensive factors on the detection of csPCA has several important findings.

First, PSA-density and DRE remain cornerstones of risk-assessment, especially in patients with high suspicion of PCA on mpMRI. This finding is in accordance with previous studies [9] and guideline recommendations [4]. Specifically, the link between PSA-density and csPCA has been reported before [10, 17]. While it is known that the addition of PSA-D to PI-RADS improves the overall predictive performance [18, 19], we validated this in a high-risk cohort of PI-RADS 5 patients.

Second, DRE is not devoid of limitations, and is prone to interobserver variability and errors [20]. However, at a high-volume center and in patients with highly suspect lesions, it achieved significant and clinically relevant predictor status (OR: 5.86, p < 0.001). For instance, in the PI-RADS 5 patients with a PSA-D between 0.2 and 0.5 ng/ml/cc, a positive DRE improved csPCA-detection from 175/200 (88%) to 57/59 (97%) (Table 2). These results confirm those of previous studies, such as the ERSPC-trial [11]. Therefore, we observed an impressive congruence of highly suspicious findings in palpation, suspect laboratory and imaging results for PCA detection. In other words, if one can feel, see, and measure a lesion suspected for cancer properly in the lab, it will be most likely a malignancy.

This research supports the biopsy-free strategy proposed by Meissner et al. for patients with high PSA in small prostates (e.g., 25 cc prostate volume with PSA of 12,5 ng/ml), even without PSMA-PET/CT [2]. Specifically, 54/54 patients with PI-RADS 5 and PSA-D > 0.5 ng/ml/cc had csPCA. In general, the potential harm of biopsies is small and potentially neglectable [21–23]. Especially patients, for whom radical prostatectomy is the preferred treatment option, are rarely unable to undergo biopsy at adequate risk. The effect of upstaging at RP due to treatment delay is in fusion biopsy rather irrelevant [24]. Biopsy results are indispensable for precise treatment planning, such as the necessity of lymphadenectomy and choosing staging/treatment modalities. While the combination of PI-RADS 5 and PSA-D > 0.5 ng/ml/cc did predict csPCA very accurately, these patients presented with a heterogeneous spectrum of Gleason grade groups. For instance, GGG 2 and GGG 5 were present in 24/54 (44%) and 10/54 (19%) patients, respectively (Table 2). In this light, the biopsy-omitting approach should be considered mainly for well-informed, locally advanced high-risk PCA patients, accepting the risk of suboptimal and often over-intensified treatment. In surgery, this might be the sacrifice of any primary nerve-sparing approach and an unnecessarily high rate of lymphadenectomies. In radiotherapy, adjuvant ADT duration may be prolonged excessively due to the uncertainties without biopsy (i.e., up to 3 years for high-risk versus 6 months for intermediate-risk patients) [4, 5]. However, direct initiation of ADT for instance appears reasonable in patients with a double high-risk constellation based on findings from the field of urology and other comorbidities, i.e. high-risk for csPCA and high-risk for biopsy-related complications. Age is a known risk factor for csPCA [25] and upstaging at RP [26] as well as for comorbidities such as cardio-vascular disease [27] with,e.g., the necessity for dual platelet inhibition. Therefore, especially in this cohort of patients the added value of a biopsy- vs. imaging-guided approach appears marginal.

The potential ability to skip biopsies in (double) high-risk constellations contrasts with the heterogeneity in the literature for omitting biopsies in PI-RADS 3 patients with a certain PSA-D cutoff [28]. Sensitivity of mpMRI as well as PSA-D increase with higher-grade, larger cancers [29, 30]. Therefore, strategies to omit biopsies based on these parameters are more reasonable on the high-suspicion end of the scale.

Third, diligent work and review of cases are of utmost importance. The simple combination of PSA-D and DRE had an AUC of 0.79 in PI-RADS 5 patients. Only 7 of 493 (1%) patients with PI-RADS 5 had normal prostatic tissue, while most negative biopsies showed some type of inflammation.

Despite its strengths, our study is not devoid of limitations. First, specimens from radical prostatectomy and long-term oncologic follow-up of negative biopsies were not available as reference standards. Second, as it is the very nature of retrospective analysis of a tertiary care referral center, the potential different levels of selection bias should not be underestimated. Furthermore, this explorative study reported data from highly experienced biopsy-operators, genitourinary radiologists, and pathologists. Especially for PSA-D calculations with limited standardization of prostate volume measurement, and heterogeneity in MRI reading and measurement within different institutions, our data may not be representative of different settings. Through advances in biopsy technologies, such as the transperineal approach, biopsies can be offered to almost all patients who are eligible for active treatment. This has and will decrease the incentive to omit the biopsy, making our research less relevant. In general, we do not propose abolishing the biopsy in "clear cases" per se, but offer the patients who wish to avoid the diagnostic delay, discomfort, and potential complications of prostate biopsy the opportunity to choose themselves.

## Conclusions

PSA-D and DRE were strong predictors of csPCA at biopsy in PI-RADS 5 patients. Therefore, these patients who have received a pathology report negative for PCA should be followed-up carefully. Conversely, biopsies may be omitted prior treatment in well-informed patients with a high suspicion of locally advanced high-risk PCA, especially if risk-factors for biopsy-related complications such as obligatory dual platelet inhibition are present. These patients, however, must accept the risk of suboptimal or overtreatment by this biopsy-free approach.

## Supplementary Information

Below is the link to the electronic supplementary material.

Supplementary file1 (DOCX 29 KB)

## Data Availability

Data is non-public but may be requested.

## Abbreviations

ADT: Androgen deprivation therapy
AUC: Area under the curve
Bx: MRI/ultrasound fusion biopsy (lesion targeted and systematic)
csPCA: Clinically significant prostate cancer, defined as GGG ≥ 2
cT-stage: Clinical T-stage according to DRE findings
DRE: Digital rectal examination
ECE: Extracapsular extension
GGG: Gleason Grade Group
IQR: Interquartile ranges
mpMRI: Multi-parametric magnetic resonance imaging
PCA: Prostate cancer
PI-RADS: Prostate imaging reporting and data system
PI-RADS 5 patients: Patients with at least one PI-RADS 5 lesion
PSA: Prostate-specific antigen
PSA-D: PSA-density
ROC: Receiver operating characteristic

## References

[1] Ljungberg B, Albiges L, Abu-Ghanem Y, Bedke J, Capitanio U, Dabestani S et al (2022) European Association of Urology Guidelines on renal cell carcinoma: the 2022 update. Eur Urol 82(4):399–410 35346519 10.1016/j.eururo.2022.03.006

[2] Meissner VH, Rauscher I, Schwamborn K, Neumann J, Miller G, Weber W et al (2022) Radical prostatectomy without prior biopsy following multiparametric magnetic resonance imaging and prostate-specific membrane antigen positron emission tomography. Eur Urol 82(2):156–160 34887117 10.1016/j.eururo.2021.11.019

[3] Emmett L, Buteau J, Papa N, Moon D, Thompson J, Roberts MJ et al (2021) The additive diagnostic value of prostate-specific membrane antigen positron emission tomography computed tomography to multiparametric magnetic resonance imaging triage in the diagnosis of prostate cancer (PRIMARY): a prospective multicentre study. Eur Urol 80(6):682–689 34465492 10.1016/j.eururo.2021.08.002

[4] Mottet N, van den Bergh RCN, Briers E, Van den Broeck T, Cumberbatch MG, De Santis M et al (2021) EAU-EANM-ESTRO-ESUR-SIOG Guidelines on Prostate Cancer-2020 update. Part 1: screening, diagnosis, and local treatment with curative intent. Eur Urol. 79(2):243–262 33172724 10.1016/j.eururo.2020.09.042

[5] Network NCC. Prostate Cancer (Version 1.2023) 2022. https://www.nccn.org/professionals/physician_gls/pdf/prostate.pdf 10.6004/jnccn.2022.0063 36509074

[6] Leitlinienprogramm Onkologie (Deutsche Krebsgesellschaft DK, AWMF):. S3-Leitlinie Prostatakarzinom, Langversion 6.0, 2021, AWMF Registernummer: 043/022OL, http://www.leitlinienprogramm-onkologie.de/leitlinien/prostatakarzinom/ 2021

[7] Kasivisvanathan V, Rannikko AS, Borghi M, Panebianco V, Mynderse LA, Vaarala MH et al (2018) MRI-targeted or standard biopsy for prostate-cancer diagnosis. N Engl J Med 378(19):1767–1777 29552975 10.1056/NEJMoa1801993 PMC9084630

[8] van der Leest M, Cornel E, Israël B, Hendriks R, Padhani AR, Hoogenboom M et al (2019) Head-to-head comparison of transrectal ultrasound-guided prostate biopsy versus multiparametric prostate resonance imaging with subsequent magnetic resonance-guided biopsy in biopsy-naïve men with elevated prostate-specific antigen: a large prospective multicenter clinical study. Eur Urol 75(4):570–578 30477981 10.1016/j.eururo.2018.11.023

[9] Washino S, Okochi T, Saito K, Konishi T, Hirai M, Kobayashi Y, Miyagawa T (2017) Combination of prostate imaging reporting and data system (PI-RADS) score and prostate-specific antigen (PSA) density predicts biopsy outcome in prostate biopsy naïve patients. BJU Int 119(2):225–233 26935594 10.1111/bju.13465

[10] Nordström T, Akre O, Aly M, Grönberg H, Eklund M (2018) Prostate-specific antigen (PSA) density in the diagnostic algorithm of prostate cancer. Prostate Cancer Prostatic Dis 21(1):57–63 29259293 10.1038/s41391-017-0024-7

[11] Gosselaar C, Roobol MJ, Roemeling S, Schröder FH (2008) The role of the digital rectal examination in subsequent screening visits in the European randomized study of screening for prostate cancer (ERSPC). Rotterdam Eur Urol 54(3):581–588 18423977 10.1016/j.eururo.2008.03.104

[12] Weinreb JC, Barentsz JO, Choyke PL, Cornud F, Haider MA, Macura KJ et al (2016) PI-RADS prostate imaging—reporting and data system: 2015, Version 2. Eur Urol 69(1):16–40 26427566 10.1016/j.eururo.2015.08.052 PMC6467207

[13] Barentsz JO, Richenberg J, Clements R, Choyke P, Verma S, Villeirs G et al (2012) ESUR prostate MR guidelines 2012. Eur Radiol 22(4):746–757 22322308 10.1007/s00330-011-2377-y PMC3297750

[14] de Rooij M, Israël B, Tummers M, Ahmed HU, Barrett T, Giganti F et al (2020) ESUR/ESUI consensus statements on multi-parametric MRI for the detection of clinically significant prostate cancer: quality requirements for image acquisition, interpretation and radiologists’ training. Eur Radiol 30(10):5404–5416 32424596 10.1007/s00330-020-06929-z PMC7476997

[15] Leyh-Bannurah SR, Boiko S, Beyersdorff D, Falkenbach F, Ekrutt J, Maurer T et al (2022) Pan-segmental intraprostatic lesions involving mid-gland and apex of prostate (mid-apical lesions): assessing the true value of extreme apical biopsy cores. World J Urol 40(7):1653–1659 35501610 10.1007/s00345-022-04006-2 PMC9236964

[16] Kasivisvanathan V, Stabile A, Neves JB, Giganti F, Valerio M, Shanmugabavan Y et al (2019) Magnetic resonance imaging-targeted biopsy versus systematic biopsy in the detection of prostate cancer: a systematic review and meta-analysis. Eur Urol 76(3):284–303 31130434 10.1016/j.eururo.2019.04.043

[17] Omri N, Kamil M, Alexander K, Alexander K, Edmond S, Ariel Z et al (2020) Association between PSA density and pathologically significant prostate cancer: the impact of prostate volume. Prostate 80(16):1444–1449 32970856 10.1002/pros.24078

[18] Stevens E, Truong M, Bullen JA, Ward RD, Purysko AS, Klein EA (2020) Clinical utility of PSAD combined with PI-RADS category for the detection of clinically significant prostate cancer. Urol Oncol. 38(11):8469.e9-e16 10.1016/j.urolonc.2020.05.024 32576527

[19] Schoots IG, Padhani AR (2021) Risk-adapted biopsy decision based on prostate magnetic resonance imaging and prostate-specific antigen density for enhanced biopsy avoidance in first prostate cancer diagnostic evaluation. BJU Int 127(2):175–178 33089586 10.1111/bju.15277 PMC7894174

[20] Gosselaar C, Kranse R, Roobol MJ, Roemeling S, Schröder FH (2008) The interobserver variability of digital rectal examination in a large randomized trial for the screening of prostate cancer. Prostate 68(9):985–993 18409186 10.1002/pros.20759

[21] Roberts MJ, Bennett HY, Harris PN, Holmes M, Grummet J, Naber K, Wagenlehner FME (2017) Prostate biopsy-related infection: a systematic review of risk factors, prevention strategies, and management approaches. Urology 104:11–21 28007492 10.1016/j.urology.2016.12.011

[22] Loeb S, Vellekoop A, Ahmed HU, Catto J, Emberton M, Nam R et al (2013) Systematic review of complications of prostate biopsy. Eur Urol 64(6):876–892 23787356 10.1016/j.eururo.2013.05.049

[23] Jacewicz M, Günzel K, Rud E, Sandbæk G, Magheli A, Busch J et al (2022) Antibiotic prophylaxis versus no antibiotic prophylaxis in transperineal prostate biopsies (NORAPP): a randomised, open-label, non-inferiority trial. Lancet Infect Dis 22(10):1465–1471 35839791 10.1016/S1473-3099(22)00373-5

[24] Kachanov M, Budäus L, Witt JH, Wagner C, Zinke J, Fangmeyer B et al (2022) Suitability of conventional systematic vs MRI-guided targeted biopsy approaches to assess surgical treatment delay for radical prostatectomy. World J Urol 40(12):2955–2961 36357604 10.1007/s00345-022-04207-9 PMC9649392

[25] Bechis SK, Carroll PR, Cooperberg MR (2011) Impact of age at diagnosis on prostate cancer treatment and survival. J Clin Oncol 29(2):235–241 21135285 10.1200/JCO.2010.30.2075 PMC3058279

[26] Richstone L, Bianco FJ, Shah HH, Kattan MW, Eastham JA, Scardino PT, Scherr DS (2008) Radical prostatectomy in men aged >or=70 years: effect of age on upgrading, upstaging, and the accuracy of a preoperative nomogram. BJU Int 101(5):541–546 18257855 10.1111/j.1464-410X.2007.07410.x

[27] Rodgers JL, Jones J, Bolleddu SI, Vanthenapalli S, Rodgers LE, Shah K et al (2019) Cardiovascular risks associated with gender and aging. J Cardiovasc Dev Dis. 6(2):19 31035613 10.3390/jcdd6020019 PMC6616540

[28] Maggi M, Panebianco V, Mosca A, Salciccia S, Gentilucci A, Di Pierro G et al (2020) Prostate imaging reporting and data system 3 category cases at multiparametric magnetic resonance for prostate cancer: a systematic review and meta-analysis. Eur Urol Focus 6(3):463–478 31279677 10.1016/j.euf.2019.06.014

[29] Johnson DC, Raman SS, Mirak SA, Kwan L, Bajgiran AM, Hsu W et al (2019) Detection of individual prostate cancer foci via multiparametric magnetic resonance imaging. Eur Urol 75(5):712–720 30509763 10.1016/j.eururo.2018.11.031

[30] Andolfi C, Vickers AJ, Cooperberg MR, Carroll PR, Cowan JE, Paner GP et al (2022) Blood prostate-specific antigen by volume of benign, gleason pattern 3 and 4 prostate tissue. Urology 170:154–160 35987380 10.1016/j.urology.2022.08.014 PMC10515713

