# Supplementary material for: PSA-density, DRE, and PI-RADS 5: potential surrogates for omitting biopsy?
Source: World J Urol. 2024 Mar 20;42(1):182. doi: 10.1007/s00345-024-04894-6 (PMC10955031; doi:10.1007/s00345-024-04894-6)
Supplement: Supplementary file 1 — Supplementary file1 (DOCX 29 KB) [file 345_2024_4894_MOESM1_ESM.docx]

**Supplementary Material**

**3441 consecutive patients** referred for prostate fusion biopsy between 2016 and 2020

**Excluded (n = 129)**

- inclusion in active surveillance protocol (n = 81)

-prior treatment to the prostate such as TUR-P (n=46)

**3314 study cohort**

- 493 PI-RADS 5

**Figure 2**: CONSORT flow diagram.

| **Models** | **OR** | **95% CI** | **p-value** | **AUC** | **95% CI** |
| --- | --- | --- | --- | --- | --- |
| **Model A** |  |  |  | 0.51 | 0.48 – 0.55 |
| ECE |  |  |  |  |  |
| No | Ref. |  |  |  |  |
| Yes | 0.60 | 0.27, 1.48 | 0.2 |  |  |
| **Model B** |  |  |  | 0.72 | 0.66 – 0.77 |
| PSA-D^1^ | 2.12 | 1.65, 2.82 | <0.001 |  |  |
| **Model C** |  |  |  | 0.63 | 0.59 – 0.67 |
| cT-stage |  |  |  |  |  |
| cT1 | Ref. |  |  |  |  |
| cT2 | 5.89 | 2.83, 14.3 | <0.001 |  |  |
| **Model D** |  |  |  | 0.79 | 0.74 – 0.83 |
| PSA-D^1^ | 2.27 | 1.72, 3.09 | <0.001 |  |  |
| cT-stage |  |  |  |  |  |
| cT1 | — | — |  |  |  |
| cT2 | 5.86 | 2.76, 14.5 | <0.001 |  |  |
|  | | | |  |  |

**Table 3:** Logistic regression models to analyze the association of the predictors with respect to clinically significant prostate cancer diagnosed at prostate biopsy in patients with PI-RADS 5 lesions at mpMRI.

OR = Odds Ratio; CI = Confidence Interval; AUC = area under the curve; Ref. = reference; ECE = extracapsular extension; PSA = prostate-specific antigen.

^1^ The predictor PSA-Density was rescaled to obtain more interpretable estimates: odd ratio per 10 units, PSA-Density was measured in ng/ml/cc.

|  | **PI-RADS 5 patients without cancer**  (n=36) |
| --- | --- |
| PSA, ng/mL (median, IQR) | 6.7 (4.9, 8.7) |
| Age, years (median, IQR) | 66 (58, 68) |
| Prostate volume (mL), median (IQR) | 58 (42, 86) |
| PSA-Density, ng/ml/cc (median, IQR) | 0.11 (0.07, 0.19) |
| <0.1 | 14 (39%) |
| <0.2 | 15 (42%) |
| <0.5 | 7 (19%) |
| ≥0.5 | 0 (0%) |
| cT-stage (n, %) |  |
| cT1 | 35 (97%) |
| cT2 | 1 (3%) |
| Rebiopsy performed (n, %) | 13 (28%) |
| csPCa at Rebiopsy (n, %) | 3 (8%) |

**Table 4:** Descriptive characteristics of 36 consecutive patients with a PI-RADS 5 lesion and a negative prostate biopsy.
